# Supplementary material for: Identifying Environmental Impact Factors for Sustainable Healthcare: A Scoping Review
Source: Int J Environ Res Public Health. 2023 Sep 12;20(18):6747. doi: 10.3390/ijerph20186747 (PMC10531011; doi:10.3390/ijerph20186747)
Supplement: Supplementary file 1 [file ijerph-20-06747-s001.zip › ijerph-2578736-supplementary.pdf]

International Journal of
Environmental Research and Public Health

Supplementary Materials

Supplementary Material S1: Scoping Review Protocol

Search query PubMed:

((“delivery of health care”[MeSH Terms] OR “health facilities”[MeSH Terms] OR “clinic”[Title/Abstract] OR “healthcare sector”[Title/Abstract] OR “health-care system”[Title/Abstract] OR “hospital”[Title/Abstract] OR “healthcare industry”[Title/Abstract]) AND (“ecological and environmental phenomena”[MeSH Terms] OR “Climate Change”[MeSH Terms] OR “Climate change”[Title/Abstract] OR (“environment”[Title/Abstract] AND “responsibility”[Title/Abstract]) OR “global warming”[Title/Abstract] OR “planetary health”[Title/Abstract] OR (“climate”[Title/Abstract] AND “sustainability”[Title/Abstract]) OR “ecological impact”[Title/Abstract] OR “environmental impact”[Title/Abstract] OR “changing climate”[Title/Abstract] OR (“climate”[Title/Abstract] AND “responsibility”[Title/Abstract])) AND (“Carbon Footprint”[MeSH Terms] OR “Effect measure”[Title/Abstract] OR “impact factor”[Title/Abstract] OR “emission”[Title/Abstract] OR “Quantities”[Title/Abstract] OR “Quantity”[Title/Abstract] OR “footprint”[Title/Abstract] OR “quantification”[Title/Abstract])).

Search query EBSCOhost databases:

AB ((clinic OR (healthcare sector) OR (health-care system) OR hospital OR (healthcare industry))) AND AB ((carbon footprint) OR (effect measure) OR (impact factor) OR emission OR quantity OR quantities OR footprint OR quantification)) AND AB ((climate change) OR (environment AND responsibility) OR (global warming) OR (planetary health) OR (climate AND sustainability) OR (ecological impact) OR (environmental impact) OR (changing climate) OR (climate AND responsibility)).

Inclusion criteria:

- Presentation of original research on measuring environmental impact;
- Reporting of one or more impact factors that represent the environmental impact of a single or group of healthcare services or activities, including the product(s) used for the service or activity;
- Describing impact factors based on primary data.

Exclusion criteria:

- Written in language other than English;
- No full article was available.

Supplementary Material S2: Overview of Environmental Impact Factors per Unit, Component or Scope and Healthcare Setting

Table S1. Overview of environmental impact factors for waste generation per unit, component or scope and healthcare setting.

Direct Pressure: Waste Generation			
**Unit**	**Component/Scope**	**Healthcare Setting**	**Source**
kg per treatment/procedure/patient	General/total waste mass	General—all	Cameron et al., 2021 [57]
		General emergency room	Hsu et al., 2020 [52]
		General operating room	Rammelkamp et al., 2021 [54]

	General operating room	MacNeill et al. (2017) [46]
	Orthopedic surgery (general)	Kooner et al., 2019 [51]
	Intravitreal injection	Rammelkamp et al., 2021 [52]
	Cystoscopy	Rammelkamp et al., 2021 [54]
	Carpal Tunnel Release	Rammelkamp et al., 2021 [54]
	Vasectomy	Rammelkamp et al., 2021 [54]
	Laryngoscopy	Rammelkamp et al., 2021 [54]
	Microscopic Cataract Surgery	Rammelkamp et al., 2021 [54]
	Transurethral Resection of Prostate	Rammelkamp et al., 2021 [54]
	Sural Nerve Biopsy	Rammelkamp et al., 2021 [54]
	Gastrocnemius Repair	Rammelkamp et al., 2021 [54]
	Debridement of Foot	Rammelkamp et al., 2021 [54]
	Biopsy of Penile Mass	Rammelkamp et al., 2021 [54]
	Deep Brain Stimulation	Rammelkamp et al., 2021 [54]
	Ureteroscopy	Rammelkamp et al., 2021 [54]
	Transurethral Resection of Prostate	Rammelkamp et al., 2021 [54]
	Radiofrequency Ablation	Rammelkamp et al., 2021 [54]
	Exam Under Anesthesia	Rammelkamp et al., 2021 [54]
	Fistulotomy	Rammelkamp et al., 2021 [54]
	Mandible Repair	Rammelkamp et al., 2021 [54]
	Open Hernia Repair	Rammelkamp et al., 2021 [54]
	Orthopedic Surgery—Foot and Ankle	Kooner et al., 2019 [51]
	Orthopedic Surgery—Ankle	Kooner et al., 2019 [51]
	Orthopedic Surgery—Facet Joint Injections	Shouthorn et al., 2013 [34]
	Orthopedic Surgery—Upper Extremity	Kooner et al., 2019 [51]
	Orthopedic Surgery—Trauma	Kooner et al., 2019 [51]
	Orthopedic Surgery—Sports	Kooner et al., 2019 [51]
	Orthopedic Sur-	Kooner et al., 2019 [51]

	gery—Arthroplasty	
	Orthopedic Surgery—Total Knee Arthroplasty	Shouthorn et al., 2013 [34]
	Orthopedic Surgery—Total Knee Arthroplasty	De Sa et al., 2016 [42]
	Orthopedic Surgery—Total Hip Arthroplasty	Shouthorn et al., 2013 [34]
	Cystoscopy + Procedure	Rammelkamp et al., 2021 [54]
	Amputation—Below Knee	Rammelkamp et al., 2021 [54]
	Skin Lesion Excision	Rammelkamp et al., 2021 [54]
	Mouth Excision	Rammelkamp et al., 2021 [54]
	Penile Implant	Rammelkamp et al., 2021 [54]
	Orchiectomy	Rammelkamp et al., 2021 [54]
	Incision and Drainage	Rammelkamp et al., 2021 [54]
	Laparoscopic Surgery—Hernia Repair	Rammelkamp et al., 2021 [54]
	Laminectomy	Rammelkamp et al., 2021 [54]
	Coronary Surgery—Transcarotid Artery Revascularization	Rammelkamp et al., 2021 [54]
	Laparoscopic Surgery—Sleeve Gastrectomy	Rammelkamp et al., 2021 [54]
	Angiogram	Rammelkamp et al., 2021 [54]
	Amputation—Guillotine	Rammelkamp et al., 2021 [54]
	Lobectomy—Video-assisted Thoracoscopy	Rammelkamp et al., 2021 [54]
	Laparoscopic Surgery—Gastric Bypass	Rammelkamp et al., 2021 [54]
	Laparoscopic Surgery—Bowel Resection	Rammelkamp et al., 2021 [54]
	Coronary Surgery—Aneurysm Repair	Rammelkamp et al., 2021 [54]
	Laparoscopic Surgery—Prostatectomy	Rammelkamp et al., 2021 [54]
	Coronary Surgery—Transcatheter Aortic Valve Replacement	Rammelkamp et al., 2021 [54]
	Coronary Surgery—Coronary Artery Bypass	Rammelkamp et al., 2021 [54]

		Orthopedic Surgery—Arthroplasty	Rammelkamp et al., 2021 [54]
		Otolaryngology—Head and Neck Surgery	Lui et al., 2014 [37]
kg per treatment/procedure/patient	General recyclable waste mass	General	Rammelkamp et al., 2021 [54]
		Emergency care	Hsu et al., 2020 [52]
		Orthopedic surgery	Kooner et al., 2019 [51]
		Otolaryngology surgery	Lui et al., 2014 [37]
		Rhinology—Endoscopic Sinus Surgery	Lui et al., 2014 [37]
		Laryngology	Lui et al., 2014 [37]
		Facial plastics	Lui et al., 2014 [37]
		Head and Neck Oncology	Lui et al., 2014 [37]
		Otology-Neurotology	Lui et al., 2014 [37]
		Pediatric Otolaryngology—Head and Neck Surgery	Lui et al., 2014 [37]
		Orthopedic Surgery—Total Knee Arthroplasty—recyclable plastics	De Sa et al., 2016 [42]
		Pediatric Otolaryngology—Head and Neck Surgery	Lui et al., 2014 [37]
	General nonrecyclable waste mass	Orthopedic surgery	Kooner et al., 2019 [51]
		Rhinology—Endoscopic Sinus Surgery	Lui et al., 2014 [37]
		Laryngology	Lui et al., 2014 [37]
		Facial plastics	Lui et al., 2014 [37]
		Head and Neck Oncology	Lui et al., 2014 [37]
		Otology-Neurotology	Lui et al., 2014 [37]
	Regulated medical waste mass	Emergency care	Hsu et al., 2020 [52]
		Neurosurgery	Talibi et al., 2022 [61]
		Coronary surgery (general)	Debois et al., 2013 [34]
	Hazardous waste mass	Surgery (general)	Rammelkamp et al., 2021 [54]
		Orthopedic Surgery—Total Knee Arthroplasty	De Sa et al., 2016 [42]
		Orthopedic surgery (general)	Kooner et al., 2019 [51]
		Interventional Radiology	Chua et al., 2021 [58]
		Intravitreal injection	Cameron et al., 2021 [57]
	Municipal solid waste mass	Interventional Radiology	Chua et al., 2021 [58]
		Emergency care	Hsu et al., 2020 [52]
		Hysterectomy—Robotic	Thiel et al., 2015 [41]
		Hysterectomy—Abdominal	Thiel et al., 2015 [41]
		Orthopedic Surgery—Total Knee Arthroplasty	De Sa et al., 2016 [42]
	Blue wrap waste mass	Surgery (general)	Rammelkamp et al., 2021 [54]
		Orthopedic Surgery—Total Knee Arthroplasty	De Sa et al., 2016 [42]

	Electronic waste mass	Emergency care	Hsu et al., 2020 [52]
	Food waste mass	Emergency care	Hsu et al., 2020 [52]
		Wards	Dias-Ferreira et al. (2015) [40]
		Otolaryngology	Dias-Ferreira et al. (2015) [40]
		Oncology	Dias-Ferreira et al. (2015) [40]
		Ophthalmology	Dias-Ferreira et al. (2015) [40]
		Nephrology	Dias-Ferreira et al. (2015) [40]
		Hematology	Dias-Ferreira et al. (2015) [40]
		Endocrinology	Dias-Ferreira et al. (2015) [40]
		Renal transplants	Dias-Ferreira et al. (2015) [40]
		Urology	Dias-Ferreira et al. (2015) [40]
		General medicine	Dias-Ferreira et al. (2015) [40]
		Gastroenterology	Dias-Ferreira et al. (2015) [40]
		Neuro surgery	Dias-Ferreira et al. (2015) [40]
		Vascular surgery	Dias-Ferreira et al. (2015) [40]
		Orthopedics	Dias-Ferreira et al. (2015) [40]
		Cardiology	Dias-Ferreira et al. (2015) [40]
		Pneumology	Dias-Ferreira et al. (2015) [40]
		Neurology	Dias-Ferreira et al. (2015) [40]
		Psychiatry	Dias-Ferreira et al. (2015) [40]
		Operating Room	Dias-Ferreira et al. (2015) [40]
	Glass waste mass	Emergency care	Hsu et al., 2020 [52]
	Laundry/textiles waste mass	Interventional Radiology	Chua et al., 2021 [58]
		Orthopedic Surgery—Total Knee Arthroplasty	De Sa et al., 2016 [42]
		Emergency care	Hsu et al., 2020 [52]
	Metals waste mass	Emergency care	Hsu et al., 2020 [52]
	Sharps waste mass	Interventional Radiology	Chua et al., 2021 [58]
		Emergency care	Hsu et al., 2020 [52]

		Orthopedic Surgery—Total Knee Arthroplasty	De Sa et al., 2016 [42]
	Packaging of products waste mass	Intravitreal injection	Cameron et al., 2021 [57]
	Paper waste mass	Intravitreal injection—paper towel	Cameron et al., 2021 [57]
		Emergency care—paper towel	Hsu et al., 2020 [52]
	Plastics waste mass	Emergency care—soft plastics	Hsu et al., 2020 [52]
		Emergency care—hard plastics	Hsu et al., 2020 [52]
Liters per treatment/procedure/patient	General/total waste volume	Otolaryngology—Head and Neck Surgery	Lui et al., 2014 [37]
	Recyclable waste volume	Otolaryngology—Head and Neck Surgery—preoperative	Lui et al., 2014 [37]
kg per day per bed/patient	General recyclable waste mass	All—private hospital	Eker & Bilgili, 2011 [26]
		All—state hospital	Eker & Bilgili, 2011 [26]
		All—university hospital	Eker & Bilgili, 2011 [26]
		All—small private clinic	Eker & Bilgili, 2011 [26]
		All—dialysis treatment center	Eker & Bilgili, 2011 [26]
	Regulated medical waste mass	All—private hospital	Eker & Bilgili, 2011 [26]
		All—state hospital	Eker & Bilgili, 2011 [26]
		All—university hospital	Eker & Bilgili, 2011 [26]
		All—small private clinic	Eker & Bilgili, 2011 [26]
		All—dialysis treatment center	Eker & Bilgili, 2011 [26]
	Hazardous waste mass	All—national	Maamari et al., 2015 [38]
		All—private hospital	Eker & Bilgili, 2011 [26]
		All—state hospital	Eker & Bilgili, 2011 [26]
		All—university hospital	Eker & Bilgili, 2011 [26]
		All—small private clinic	Eker & Bilgili, 2011 [26]
		All—dialysis treatment center	Eker & Bilgili, 2011 [26]
	Municipal solid waste mass	All—private hospital	Eker & Bilgili, 2011 [26]
		All—state hospital	Eker & Bilgili, 2011 [26]
		All—university hospital	Eker & Bilgili, 2011 [26]
		All—small private clinic	Eker & Bilgili, 2011 [26]
		All—dialysis treatment center	Eker & Bilgili, 2011 [26]
	Liquid waste mass	All—private hospital	Eker & Bilgili, 2011 [26]
		All—state hospital	Eker & Bilgili, 2011 [26]
		All—university hospital	Eker & Bilgili, 2011 [26]
		All—small private clinic	Eker & Bilgili, 2011 [26]
		All—dialysis treatment center	Eker & Bilgili, 2011 [26]
	Sharps mass	All—private hospital	Eker & Bilgili, 2011 [26]
		All—state hospital	Eker & Bilgili, 2011 [26]
		All—university hospital	Eker & Bilgili, 2011 [26]
		All—small private clinic	Eker & Bilgili, 2011 [26]
		All—dialysis treatment center	Eker & Bilgili, 2011 [26]
kg per day per HC center	General/total waste mass	All—small clinic	Khan et al., 2019 [49]
	Regulated medi-	All—small clinic	Khan et al., 2019 [49]

	cal waste mass		
	Hazardous waste mass	All—small clinic	Khan et al., 2019 [49]
	Municipal solid waste mass	All—private hospital	Riedel, 2011 [27]
	Paper mass	All—private hospital	Riedel, 2011 [27]
	Sharps mass	All—small clinic	Khan et al., 2019 [49]
kg CO2 or CO2, eq per treatment/ procedure	General/ total waste emission	Cataract surgery	Morris et al., 2013 [32]
		Peritoneal dialysis—at home	Chen et al., 2017 [44]
		Peritoneal dialysis—in center	Chen et al., 2017 [44]
	Regulated medical waste emission	Coronary surgery—general	Grinberg et al., 2021 [61]
kg per year per HC center	General/ total waste mass	All operating rooms for entire hospital	MacNeill et al., 2017 [46]
	Municipal solid waste mass	All operating rooms for entire hospital	MacNeill et al., 2017 [46]
	Hazardous waste mass	Laparoscopic surgery—laparoscopic trocar	Power et al., 2012 [29]
		Laparoscopic surgery—plastics	Power et al., 2012 [29]
		All operating rooms entire hospital	MacNeill et al., 2017 [46]
		Cytotoxic waste all operating rooms for entire hospital	MacNeill et al., 2017 [46]
	Mass of recyclable waste	All operating rooms for entire hospital	MacNeill et al., 2017 [46]
	Mass of waste transported	All operating room waste transported for entire hospital	MacNeill et al., 2017 [46]
	Mass of sharps waste	All operating rooms for entire hospital	MacNeill et al., 2017 [46]
	Mass of waste from re-usable textiles	All operating rooms for entire hospital	MacNeill et al., 2017 [46]
kg CO2or CO2eq per year	Emission of General/Total waste	All renal services entire healthcare center	Connor et al., 2010 [24]
		Haemo- & peritoneal dialysis entire healthcare center	Connor et al., 2010 [24]
		Outpatient visits dialysis treatment entire healthcare center	Connor et al., 2010 [24]
		Inpatient visits dialysis treatment entire healthcare center	Connor et al., 2010 [24]
		All operating rooms total waste emission	MacNeill et al., 2017 [46]
	Emission from recyclable waste	All operating rooms for entire hospital	MacNeill et al., 2017 [46]
	Emission from hazardous waste	All operating rooms for entire hospital	MacNeill et al., 2017 [46]
		All operating rooms Cytotoxic	MacNeill et al., 2017 [46]

	waste		
	Emission from municipal solid waste	All operating rooms for entire hospital	MacNeill et al., 2017 [46]
	Sharps waste emission	All operating rooms for entire hospital	MacNeill et al., 2017 [46]
	Emission from reusable textiles waste	All operating rooms for entire hospital	MacNeill et al., 2017 [46]
	Emission from transport of waste	All operating room waste transport for entire hospital	MacNeill et al., 2017 [46]

Table S2. Overview of environmental impact factors for pollutant emission per unit, component or scope and healthcare setting.

**Direct Pressure: Pollutant Emission**			
**Unit**	**Component/Scope**	**Healthcare Setting**	**Source**
		Air pollution	
kg CO_2 or CO_2eq per treatment	Pharmaceuticals (incl. anesthetics)	Coronary surgery intravenous anesthetics	Grinberg et al., 2021 [61]
		Coronary surgery sefoflurane anesthetics	Grinberg et al., 2021 [61]
		General surgery—gas anesthetics	MacNeill et al., 2017 [46]
		Cataract surgery—pharmaceuticals	Morris et al., 2013 [32]
	Full lifecycle care pathway	Wrist and Radius Injury Surgery	Baxter et al., 2021 [59]
	Transport/travel	Interventional Radiology—staff	Chua et al., 2021 [58]
		Abdominoplasty—patient	Berner et al., 2017 [47]
		Abdominoplasty—staff	Berner et al., 2017 [47]
		Breast augmentation—patient	Berner et al., 2017 [47]
		Breast augmentation—staff	Berner et al., 2017 [47]
		Rhinoplasty—patient	Berner et al., 2017 [47]
		Rhinoplasty—staff	Berner et al., 2017 [47]
		Peritoneal Dialysis—home	Chen et al., 2017 [44]
		Acute myocardial infarction—ambulance	Zander et al., 2011 [25]
		Primary Percutaneous Coronary Intervention—ambulance	Zander et al., 2011 [25]
		Mental healthcare therapy	Maughan et al., 2016 [43]
		Cataract surgery—patient	Morris et al., 2013 [32]
		Cataract surgery –staff	Morris et al., 2013 [32]
	Laundry	Abdominoplasty	Berner et al., 2017 [47]
		Breast augmentation	Berner et al., 2017 [47]
		Rhinoplasty	Berner et al., 2017 [47]
		Peritoneal Dialysis—home	Chen et al., 2017 [44]
		Peritoneal Dialysis—in center	Chen et al., 2017 [44]

		Cataract surgery	Morris et al., 2013 [32]
	General medical waste	Abdominoplasty	Berner et al., 2017 [47]
		Breast augmentation	Berner et al., 2017 [47]
		Rhinoplasty	Berner et al., 2017 [47]
	Paper use	Peritoneal Dialysis—home—towel	Chen et al., 2017 [44]
		Peritoneal Dialysis—in center—towel	Chen et al., 2017 [44]
		Peritoneal Dialysis—home—paper and ink	Chen et al., 2017 [44]
		Peritoneal Dialysis—in center—paper and ink	Chen et al., 2017 [44]
		Cataract surgery—paper and ink)	Morris et al., 2013 [32]
	Products used	Interventional Radiology—disposable supplies used	Chua et al., 2021 [58]
		Mental healthcare therapy	Maughan et al., 2016 [43]
		Cataract surgery—ICT	Morris et al., 2013 [32]
		Cataract surgery—Food	Morris et al., 2013 [32]
		Cataract surgery—Medical equipment	Morris et al., 2013 [32]
		Anesthetic gas cylinder	Gilliam et al., 2008 [19]
	Packaging of products	Peritoneal Dialysis—home	Chen et al., 2017 [44]
		Peritoneal Dialysis—in center	Chen et al., 2017 [44]
kg CO2 or CO2eq per year	Region	National medication prescription UK	Wilkinson et al., 2019 [50]
		National transport of gas for Laparoscopic surgery	Power et al., 2012 [29]
		National Laparoscopic surgery—extraction and production of gas	Power et al., 2012 [29]
		National Laparoscopic surgery use of gas	Power et al., 2012 [29]
	All in Healthcare Center	Operating room—desflurane	Patel & Smith-Steinert, 2021 [56]
		Operating room—anesthetic gas	MacNeill et al., 2017 [46]
	Transport/ travel	All renal services—patient	Connor et al., 2010 [24]
		All renal services—staff	Connor et al., 2010 [24]
		Haemo-& peritoneal dialysis—patient	Connor et al., 2010 [24]
		Outpatient visits dialysis treatment—patient	Connor et al., 2010 [24]
		Outpatient visits dialysis treatment—staff	Connor et al., 2010 [24]
		Inpatient visits dialysis treatment—patient	Connor et al., 2010 [24]
		Inpatient visits dialysis treatment—staff	Connor et al., 2010 [24]
	Pharmaceuticals	All renal services	Connor et al., 2010 [24]

	Products/Food	All renal services—Food	Connor et al., 2010 [24]
		All renal services—paper use	Connor et al., 2010 [24]
	Laundry	All renal services	Connor et al., 2010 [24]
kg CO2 or CO2eq per hour	Pharmaceuticals (incl. anesthetics)	Automated anesthetics	Tay et al., 2013 [31]
		Manual anesthetics	Tay et al., 2013 [31]
kg CO2eq per device lifetime	Products	Reusable sharps container	Grimmond & Reiner, 2012 [28]
		Disposable sharps container	Grimmond & Reiner, 2012 [28]
		Reusable laryngeal mask airways	Eckelman et al., 2020 [3]
		Disposable laryngeal mask airways	Eckelman et al., 2020 [3]
Water pollution			
μg/L continuous sampling	Pharmaceuticals	Antibiotics—Sulfamethoxazole	Diwan et al., 2013 [36]
		Antibiotics—Metronidazole	Diwan et al., 2013 [36]
		Antibiotics—Fluroquinolones	Diwan et al., 2013 [36]
		Antibiotics—Norfloxacin	Diwan et al., 2013 [36]
		Antibiotics—Ofloxacin	Diwan et al., 2013 [36]
		Antibiotics—Levofloxacin	Diwan et al., 2013 [36]
		Antibiotics—Ciprofloxacin	Diwan et al., 2013 [36]
μg/L per grasp sample	Pharmaceuticals	Antibiotics—Amoxycillin	Jha et al., 2017 [45]
		Antibiotics—Amoxycillin	Gros et al., 2013 [33]
		Antibiotics—Ampicillin	Jha et al., 2017 [45]
		Antibiotics—Azithromycin	Gros et al., 2013 [33]
		Antibiotics—Cefotaxime	Gros et al., 2013 [33]
		Antibiotics—Cephalexin	Jha et al., 2017 [45]
		Antibiotics—Chloramphenicol	Jha et al., 2017 [45]
		Antibiotics—Ciprofloxacin	Seifrtová et al., 2008 [20]
		Antibiotics—Ciprofloxacin	Jha et al., 2017 [45]
		Antibiotics—Ciprofloxacin	Diwan et al., 2013 [36]
		Antibiotics—Ciprofloxacin	Gros et al., 2013 [33]
		Antibiotics—Clarithromycin	Gros et al., 2013 [33]
		Antibiotics—Clindamycin	Gros et al., 2013 [33]
		Antibiotics—Dexamethasone	Jha et al., 2017 [45]
		Antibiotics—Enrofloxacin	Seifrtová et al., 2008 [20]
		Antibiotics—Flumequine	Gros et al., 2013 [33]
		Antibiotics—Fluroquinolones	Diwan et al., 2013 [36]
		Antibiotics—Gentamicin Sulfate	Jha et al., 2017 [45]
		Antibiotics—Levofloxacin	Jha et al., 2017 [45]
		Antibiotics—Levofloxacin	Diwan et al., 2013 [36]
		Antibiotics—Lincomycin	Gros et al., 2013 [33]
		Antibiotics—Metronidazole-OH	Gros et al., 2013 [33]
		Antibiotics—Metronidazole	Jha et al., 2017 [45]
		Antibiotics—Metronidazole	Diwan et al., 2013 [36]

		Antibiotics—Metronidazole	Gros et al., 2013 [33]
		Antibiotics—Nalidixic Acid	Gros et al., 2013 [33]
		Antibiotics—Norfloxacin	Seifrtová et al., 2008 [20]
		Antibiotics—Norfloxacin	Jha et al., 2017 [45]
		Antibiotics—Norfloxacin	Diwan et al., 2013 [36]
		Antibiotics—Norfloxacin	Gros et al., 2013 [33]
		Antibiotics—Ofloxacin	Seifrtová et al., 2008 [20]
		Antibiotics—Ofloxacin	Diwan et al., 2013 [36]
		Antibiotics—Ofloxacin	Gros et al., 2013 [33]
		Antibiotics—Penicillin	Jha et al., 2017 [45]
		Antibiotics—Sulfamethoxazole	Jha et al., 2017 [45]
		Antibiotics—Sulfamethoxazole	Diwan et al., 2013 [36]
		Antibiotics—Sulfamethoxazole	Gros et al., 2013 [33]
		Antibiotics—Sulfapyridine	Gros et al., 2013 [33]
		Antibiotics—Tetramycin	Jha et al., 2017 [45]
		Antibiotics—Trimethoprim	Gros et al., 2013 [33]
		Anti-cancer drug—2-DOH-DiF	De Oliveira Klein et al., 2021 [53]
		Anti-cancer drug—3NH2-F	De Oliveira Klein et al., 2021 [53]
		Anti-cancer drug—5-FU	De Oliveira Klein et al., 2021 [53]
		Anti-cancer drug—CP	De Oliveira Klein et al., 2021 [53]
		Anti-cancer drug—GEM	De Oliveira Klein et al., 2021 [53]
		Drug—6-Acetylcodeine	Lin et al., 2010 [22]
		Drug—6-Acetylmorphine	Lin et al., 2010 [22]
		Drug—Benzoylecgonine	Lin et al., 2010 [22]
		Drug—Cocaine	Lin et al., 2010 [22]
		Drug—Codeine	Lin et al., 2010 [22]
		Drug—Fentanyl	Lin et al., 2010 [22]
		Drug—Flunitrazepam	Lin et al., 2010 [22]
		Drug—Ketamine	Lin et al., 2010 [22]
		Drug—Methamphetamine	Lin et al., 2010 [22]
		Drug—Morphine	Lin et al., 2010 [22]
	Metals	Ag (Silver)<br>Gd (Gadolinium)<br>Pt (Platinum)	Goullé et al., 2012 [30]<br>Goullé et al., 2012 [30]<br>Goullé et al., 2012 [30]
μg/L per week	Pharmaceuticals	Glutaraldehyde	Jolibois et al., 2002 [18]
kg per year	General waste water	Operating rooms hospital	MacNeill et al., 2017 [46]
kg CO2eq per year	General waste water	Operating rooms hospital	MacNeill et al., 2017 [46]

Table S3. Overview of environmental impact factors for resource use per unit, component or scope and healthcare setting.

Direct Pressure: Resource Use			
**Unit**	**Compo-nent/Scope**	**Healthcare Setting**	**Source**
Energy use			
kg CO2eq per treat-ment	Cradle to gate (before surgery)	Elective Spinal Surgery	Wang et al., 2021 [60]
	General energy use	Coronary surgery	Grinberg et al., 2021 [61]
		Videoconference of 1 h	Masino et al., 2010 [23]
	HVAC sys-tem—use	Interventional Radiology	Chua et al., 2021 [58]
		Abdominoplasty	Berner et al., 2017 [47]
		Breast Augmentation	Berner et al., 2017 [47]
		Rhinoplasty	Berner et al., 2017 [47]
	General energy use building	Cataract Surgery	Morris et al., 2013 [32]
		Peritoneal Dialysis	Chen et al., 2017 [44]
		Mental Healthcare Therapy	Maughan et al., 2016 [43]
	General energy use at home	Peritoneal Dialysis	Chen et al., 2017 [44]
	Medical equip-ment—usage	Abdominoplasty	Berner et al., 2017 [47]
		Breast Augmentation	Berner et al., 2017 [47]
		Rhinoplasty	Berner et al., 2017 [47]
		MRI-scan incl. standby	Esmaeli et al., 2017 [48]
kg CO^{2}2eq per year	General energy use building (Di-alysis Treatment Center)	All renal services	Connor et al., 2010 [24]
		Hemo- and Peritoneal Dialy-sis	Connor et al., 2010 [24]
		Outpatient visits	Connor et al., 2010 [24]
		Inpatient visits	Connor et al., 2010 [24]
GJ per m^2	HVAC sys-tem—production and transport	Primary Healthcare Facility	Garcia-Sanz-Calcedo et al., 2021 [55]
	General energy use building	Operating room	MacNeill et al., 2017 [46]
		Entire hospital	MacNeill et al., 2017 [46]
kWh per treatment	Medical equip-ment—use	MRI-scan without standby	Esmaeli et al., 2017 [48]
		MRI-scan standby	Esmaeli et al., 2017 [48]
	HVAC sys-tem—use	Handwashing	Somner et al., 2008 [21]
kWh per hour	PC and monitor use	Standby PC and Monitor (Desktop)	McCarthy et al., 2014 [38]
MWh per m^2 per year	HVAC sys-tem—use	Operating room	MacNeill et al., 2017 [46]
MWh per year	General energy use building	Operating room	MacNeill et al., 2017 [46]
Water use			
Liters per treatment	-	Peritoneal Dialysis	Chen et al., 2017 [44]
		Handwashing—elbow on tap	Somner et al., 2008 [21]
		Handwashing—knee on tap	Somner et al., 2008 [21]
kg CO2eq per treat-ment	Fresh water use	Cataract surgery	Morris et al., 2013 [32]

kg CO2eq per year	General water use building (Dialysis Treatment Center)	All renal services	Connor et al., 2010 [24]
Mixed resource use			
kg CO2/m²	HVAC system—production and transport	Primary Healthcare Facility	Garcia-Sanz-Calcedo et al., 2021 [55]

Abbreviations: HVAC = Heating, Ventilation and Air Conditioning.
